# Feasibility Study of EO SARs as Opportunity Illuminators in Passive Radars: PAZ-Based Case Study

**DOI:** 10.3390/s151129079

**Published:** 2015-11-17

**Authors:** Jose-Luis Bárcena-Humanes, Pedro-José Gómez-Hoyo, Maria-Pilar Jarabo-Amores, David Mata-Moya, Nerea De-Rey-Maestre

**Affiliations:** Signal Theory and Communications Department, Higher Polytechnic School, University of Alcalá, Alcalá de Henares, Madrid 28805, Spain; E-Mails: jose.barcena@uah.es (J.-L.B.-H.); pedrojose.gomez@uah.es (P.-J.G.-H.); david.mata@uah.es (D.M.-M.); nerea.rey@uah.es (N.D.-R.-M.)

**Keywords:** passive radar, bistatic geometry, resolution, opportunity illuminator, synthetic aperture radar, PAZ

## Abstract

Passive radars exploit the signal transmitted by other systems, known as opportunity illuminators (OIs), instead of using their own transmitter. Due to its almost total invulnerability to natural disasters or physical attacks, satellite OIs are of special interest. In this line, a feasibility study of Earth Observation Synthetic Aperture Radar (EO SAR) systems as OIs is carried out taking into consideration signal waveform, availability, bistatic geometry, instrumented coverage area and incident power density. A case study based on the use of PAZ, the first Spanish EO SAR, is presented. PAZ transmitted waveform, operation modes, orbit characteristics and antenna and transmitter parameters are analyzed to estimate potential coverages and resolutions. The study concludes that, due to its working in on-demand operating mode, passive radars based on PAZ-type illuminators can be proposed as complementing tools during the sensor commissioning phase, for system maintenance and for improving its performance by providing additional information about the area of interest and/or increasing the data updating speed, exploiting other sensors during the time PAZ is not available.

## 1. Introduction

A passive radar (PR) is defined as a sensor whose main objective is to detect targets and to estimate parameters, such as position and speed, using non-cooperative transmitters, known as opportunity illuminators (OIs), rather than a dedicated one [1]. Broadcast, communications, radar or radio-navigation signals can be used as OIs. PRs present many advantages over active radars:

- Low development, implementation and maintenance costs.
- Easy deployment without any particular power requirement, using solar panels and batteries, and without complex civil engineering.
- Small size and low weight.
- Low probability of intercept (LPI).
- Invulnerability against the progressive erosion of communication systems, which are demanding the use of traditional radar frequencies.
- Avoidance of electromagnetic compatibility or environment impact problems.

In active radars, transmitters are usually a large fraction of the radar system cost and design effort and typically require a major share of system prime power and maintenance. The absence of their own transmitter is the main advantage of passive radars from the cost point of view. Of course, the use of non-controlled transmitters that have not been designed for radar purposes makes detection and tracking really complex. Thanks to the possibility of using commercial off-the-shelf (COTS) devices for signal reception (antenna, RF front-end and acquisition systems), an intense research activity has been carried out by research institutions, companies and universities. Technological advances, such as platforms for field-programmable gate array (FPGAs) programming or graphics processing units (GPUs), allow the reduction of the gap between technological demonstrators and prototypes. Actual active radar designs try to move the analog-to-digital conversion stage as close as possible to the antenna. Under this software-defined radio-receiving architecture, active and passive radars’ development costs can be comparable, but the active system will require its own transmitter.

Due to the high availability of satellite OIs and their almost total invulnerability to natural disasters or physical attacks, these OIs are of special interest. A deep study of the potential OIs is required, because most of the defining characteristics of the resulting PR system are inherited from the characteristics of the OI, in the same way that the behavior of an active radar is determined by the characteristics of its dedicated transmitter.

Geostationary satellites, constellations of medium Earth orbit satellites or low Earth orbit satellites have different features with respect to propagation losses, transmitted powers, central frequencies, bandwidths, waveforms, visibility times and geometry variation with time.

Systems based on geostationary communication satellites, Global Navigation Satellite System (GNSS) constellations or Earth Observation (EO) systems have been considered [2,3,4,5]. EO SARs present potential advantages associated with the use of radar signals: frequencies characterized by low atmospheric gas absorption and rain attenuation, high radiated powers, which have been designed for fulfilling radar requirements in a monostatic configuration, and low orbits, which reduce the transmitter-receiver and transmitter-target distances compared to other satellite constellations.

Synthetic Aperture Radar (SAR) sensors are key pieces of the Earth observation European Program Copernicus, previously known as GMES (Global Monitoring for Environment and Security) [6]. These sensors produce high-resolution remote-sensing imagery using antennas installed aboard mobile platforms, such as aircraft or spacecrafts. Platform movement is used to improve azimuth resolution, through the generation of a larger synthetic antenna. SAR data are used for mapping terrain and sea surfaces and detecting and classifying point and extended targets [7].

**Figure 1:**
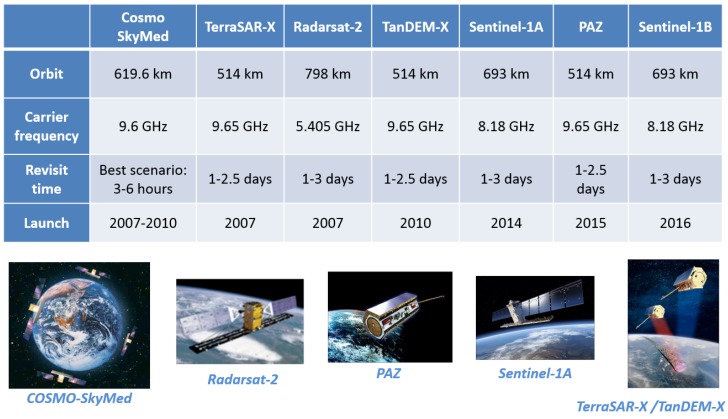
Operating SAR EO satellites’ main characteristics [8].

The high interest in these sensors has resulted in the launch of several satellites, and more launches are scheduled for the foreseeable future. Among these satellites are TerraSAR-X and next generation missions, Cosmo SkyMed, Sentinel-1 and the Radarsat mission (Figure 1) [8]. The Spanish National Earth Observation Program (PNOTS, Programa Nacional de Observación por Satélite ) has developed the PAZ system, an X-band SAR instrument mounted on a TerraSAR-X-like platform [9], to fulfill the objective of providing a constellation of observation satellites to cover Spanish EO needs and to contribute to the Copernicus and Global Earth Observation System of Systems (GEOSS) programs. PAZ is a dual system: civilian and defense. Its potential applications are those considered in Copernicus, whose main goal is to deliver products and services to manage and protect the environment and natural resources and to ensure civil security. Six main areas are considered: maritime environment, land environment, atmospheric monitoring, emergency management, security and climate change monitoring. PAZ will be operational in 2016.

Over the last decade, bistatic SAR (BSAR) techniques have been developed using spaceborne, airborne or even fixed platforms [10,11,12,13]. Space-surface BSAR (SS-BSAR), or hybrid BSAR, combines a spaceborne platform and another platform located on or near the Earth’s surface. The spaceborne platform can be a SAR sensor [10,14,15,16,17]. Some of the advantages associated with the use of bistatic systems are the following:

- Alternative geometries.
- Different scattering mechanisms.
- Ground resolution comparable to that of a monostatic system in fixed-receiver bistatic systems [16].
- Advantageous sensitivity requirements due to the shorter distance from target to receiver.

The use of a ground-based receiver allows [18]:

- Low cost experimentation of single and multichannel techniques (only a passive antenna with multiple sub-apertures with no space qualification).
- No data-link/data storage limitations.
- Data immediately available at the ground receiver.

Although the number of available SAR EO constellations is constantly growing (Figure 1), increasing the chances that an area of interest is illuminated by an EO sensor, the orbit properties (revisit time) and on-demand operating principle limit the availability of an illuminating signal. This is an important constraint, but the high cost associated with the development and launch of an EO SAR and its limited lifetime justify the study of applications based on the signal generated from space.

Passive BSAR systems have been the object of an intense research activity [19,20,21,22,23,24,25,26]. The fact that they exploit OIs instead of a controlled transmitter allows the use of other satellite illuminators (other EO sensors, GNSS or communication systems) to provide a continuous monitoring of areas of interest, instead of being limited by the revisit time of one EO satellite or constellation.

In this paper, a different approach is considered. The objective is to study the feasibility of the detection and tracking of moving targets using EO SAR satellites as OIs. Taking into consideration that the objective of passive BSAR systems is to generate radar images of Earth’s surface for detecting and classifying point and extended targets, both systems could be used as valuable complements of actual active EO SAR systems. More specifically, PRs based on these OIs could provide additional information during the EO SAR sensor calibration and operation stages. The EO SAR and the PR sensors use the same transmitted signal, so novel complementary calibration techniques can be designed. Furthermore, information retrieval from SAR images can be improved. In the first approach, PR could estimate the speed and trajectory of moving targets or provide new target features due to the bistatic geometry. The availability of commercial receiving stations makes the deployment of PRs based on EO SAR sensors in areas of special interest with a low cost feasible. Because of that, a preliminary feasibility study for a PR using the signal transmitted by PAZ is presented.

This paper is structured as follows: In Section 1, the motivation and objectives of the paper are presented; the performance principle of PRs is summarized in Section 2, and the basic parameters related to resolution and coverage are analyzed taking into consideration the bistatic geometry and the use of satellite illuminators. Section 3 describes the main features of PAZ and provides the required information for the feasibility study. Finally, Section 4 and Section 5 deal with the feasibility study and the analysis of a case study, respectively. Conclusions are presented in Section 6.

## 2. Passive Radar Performance Principle

**Figure 2:**
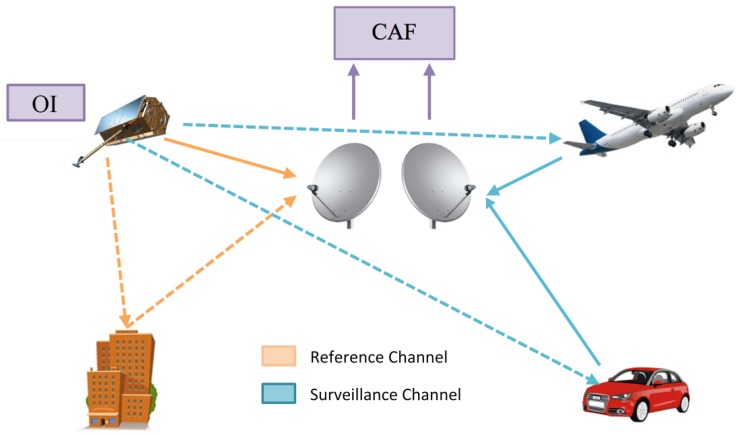
Example of passive radar geometry.

In Figure 2, the basic geometry of a bistatic PR using an EO SAR sensor as the OI is presented. The system principle of operation is based on the correlation of the reference signal from the OI acquired by the reference channel (continuous orange arrow) and the target echoes acquired by the surveillance channel (continuous blue arrows). As a result of this coherent processing, the cross-ambiguity function (CAF) is generated.

In Equation (1) the discrete time expression of the CAF is presented for a coherent integration time, $T_{int}$ (s), and a sampling frequency, $f_{s}$ (Hz), where:

- $N=T_{int}\cdot f_{s}$ is the number of samples.
- *m* represents the time bin associated with a delay $\tau_{m}=\frac{m}{f_{s}}$.
- *p* is the Doppler bin corresponding to Doppler shift $f_{D_{p}}=f_{s}\cdot \left(\frac{p}{N}\right)$.
- $s_{REF}\left[n\right]$ and $s_{SURV}\left[n\right]$ are the reference and surveillance signals, respectively.

(1) $$s_{CAF}\left[m,p\right]=\sum_{n=0}^{N-1}s_{REF}^{*}\left[n-m\right]\cdot s_{SURV}\left[n\right]\cdot \exp\left(-j2\pi \frac{p}{N}n\right)$$

**Figure 3:**
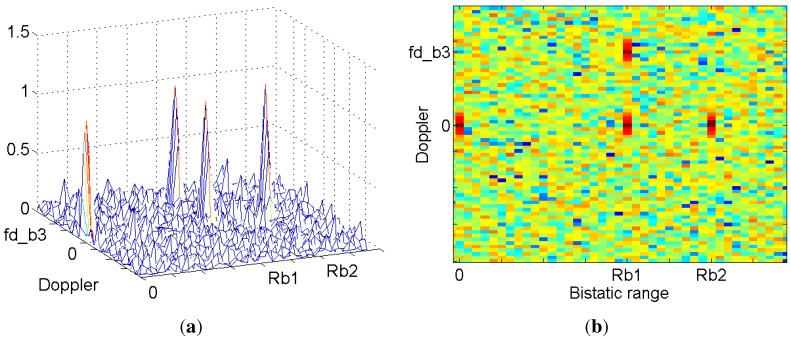
Example of the cross-ambiguity function (CAF) generated in a scenario with three targets. The effect of the DPI signal is also shown: 3D (**a**) and 2D (**b**).

For each target of the coverage area, the result of the CAF is the ambiguity function (AF) of the transmitted signal, scaled and shifted to be centered on the time delay and Doppler shift corresponding to the bistatic range and bistatic Doppler shift of the target. Figure 3 presents an example of CAF generated in a scenario with the following elements:

- Two stationary targets are at bistatic ranges Rb1 and Rb2. These bistatic ranges are calculated as $Rb=\tau_{bistatic}\cdot c$, where $\tau_{bistatic}$ is the bistatic delay calculated in Equation (2) as a function of the target-OI, the target-PR and the OI-PR or baseline distances, denoted as $R_{T},R_{R}$ and *L*, respectively. *c* is the velocity of light. Stationary targets appear in the zero Doppler line of the range-Doppler map.
  (2) $$\tau_{bistatic}=\frac{R_{T}+R_{R}}{c}-\frac{L}{c}$$
- One moving target is detected at a bistatic range Rb1. Its echo appears in the range-Doppler map at $\left(Rb1,fd_{b3}\right)$, where $fd_{b3}$ is the bistatic Doppler generated by target movement relative to the OI and the PR.
- The direct signal transmitted by the OI is captured by the reference and surveillance channels. The surveillance antenna is designed for rejecting this direct signal, but as it can be 100–80 dB higher than the target radar echoes, the level captured by the surveillance antenna can be significant compared to the target echo ones. This signal, known as the direct path interference (DPI) signal, correlates perfectly with the reference antenna signal, and as a result, a peak appears in the range-Doppler map of the CAF, located at zero bistatic range and zero Doppler.

In the following subsections, the main parameters and tools that will be used in the feasibility study are presented: the bistatic range resolution, the bistatic Doppler resolution and the bistatic radar equation.

### 2.1. Bistatic Range Resolution

**Figure 4:**
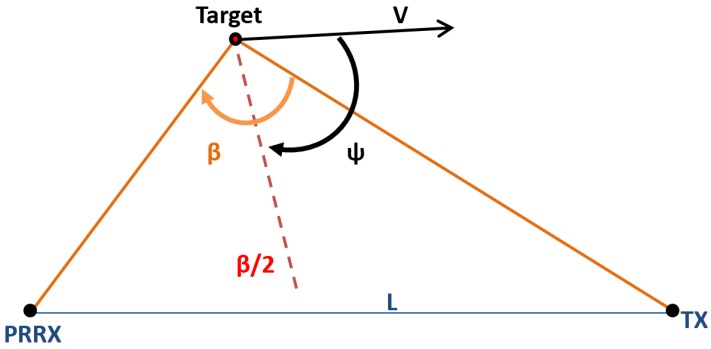
Geometry for bistatic range resolution calculation.

The bistatic range resolution, ΔR, is defined as the minimum distance between two targets that guarantees a time delay between their respective radar echoes equal to the radar compressed pulse width, $\tau_{c}$. It is calculated using Equation (3), where *B* is the signal bandwidth, $B=\frac{1}{\tau_{c}}$, *β* is the bistatic angle, *ψ* is the aspect angle with respect to the bistatic bisector and *L* is the OI-to-PR or baseline length (Figure 4). The value obtained for ψ=0 is usually used for specifying the bistatic range resolution of a system as a function of the bistatic angle.

(3) $$\Delta R=\frac{c}{2B\cos\left(\beta {/}_{2}\right)\cos\left(\psi \right)}$$

### 2.2. Bistatic Doppler Resolution

**Figure 5:**
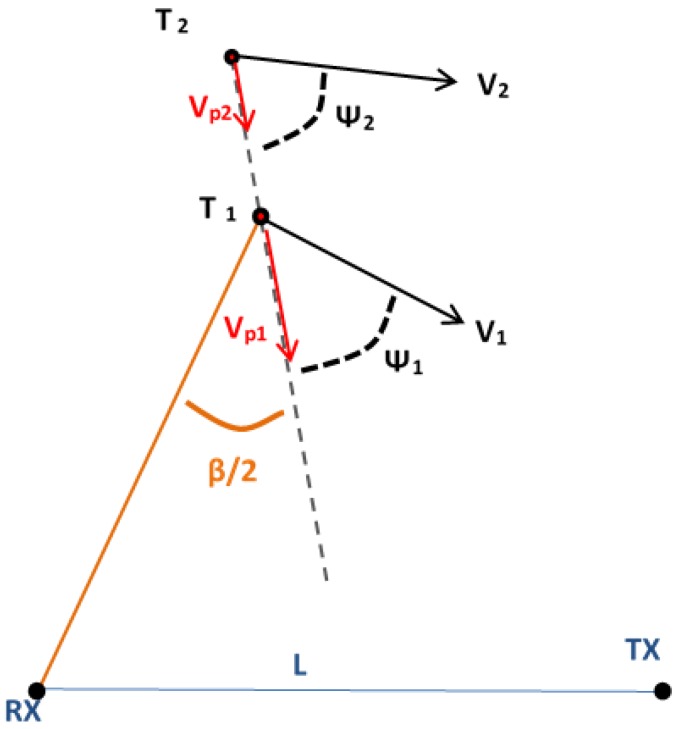
Geometry for Doppler resolution calculus.

For monostatic and bistatic Doppler resolutions, $1{/}_{T_{int}}$ is conventionally considered as an adequate Doppler separation between two target echoes at the receiver, where $T_{int}$ is the coherent integration time. In Figure 5, two targets sharing the same bistatic bisector are represented, as well as their speed component along the bistatic bisector. The required $\Delta V=|V_{P1}-V_{P2}|$ is given by expression Equation (4) [1]:

(4) $$\Delta V=\frac{\lambda }{2T_{int}\cos\left(\beta {/}_{2}\right)}$$

### 2.3. Bistatic Radar Equation

The radar equation is a mathematical expression that relates the radar range at which the specific performance is obtained to the parameters that characterize the radar (transmitted power, antenna gains, operation frequency, *etc.*), the desired target (radar cross-section (RCS)) and the radar scenario (bistatic geometry, propagation losses, multipath, clutter).

For the bistatic geometry depicted in Figure 2, the power scattered by a target can be calculated as:

(5) $$P_{R}=\frac{P_{T}G_{T}G_{R}\lambda^{2}\sigma_{bis}}{{\left(4\pi \right)}^{3}{\left(R_{R}R_{T}\right)}^{2}}\cdot A$$

where $P_{R}$ is the received power, $P_{T}$ is the power transmitted by the opportunity emitter, $G_{T}$ and $G_{R}$ are the transmitter and receiver antenna gains, respectively, *λ* is the signal wavelength, *A* represents the total propagation losses and $R_{T}$ and $R_{R}$ are the target-to-transmitter and target-to-receiver distances, respectively. The bistatic RCS, $\sigma_{bis}$, models the power scattered by the target towards the PR when it is illuminated by the OI.

### 2.4. System Coverage Limited by Sensitivity

Expression Equation (5) can be re-written as in Equation (6). The loci corresponding to $R_{T}\cdot R_{R}=constant$ is known as the oval of Cassini. For a required system sensitivity, $P_{Rmin}$, a value of $R_{R}\cdot R_{T}$ is obtained.

(6) $${\left(R_{R}R_{T}\right)}^{2}=\frac{P_{T}G_{T}G_{R}\lambda^{2}\sigma_{bis}}{{\left(4\pi \right)}^{3}P_{R}}\cdot A$$

**Figure 6:**
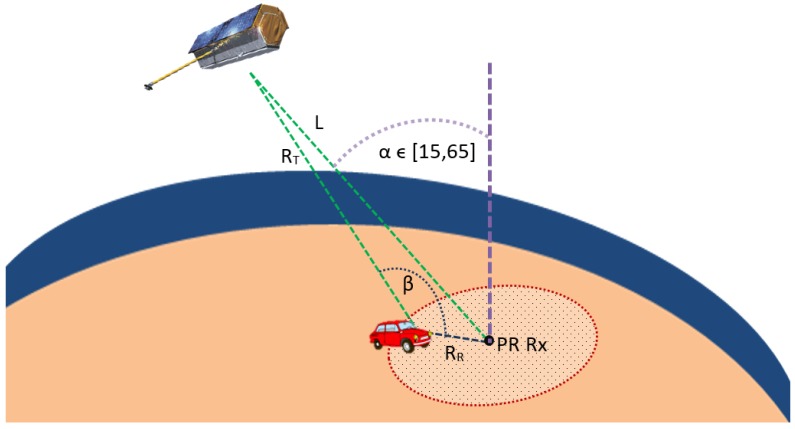
Geometry of the PAZ-based passive radar: *β* is the bistatic angle and *α* is the incidence one.

In the considered bistatic geometry, the passive radar is located on the Earth’s surface (Figure 6). Assuming a baseline length L=500 km and $R_{R}<20$ km, $L/(R_{T}\cdot R_{R})>2$, and the Cassini oval breaks into two parts, one centered on the OI and the other on the PR.

**Figure 7:**
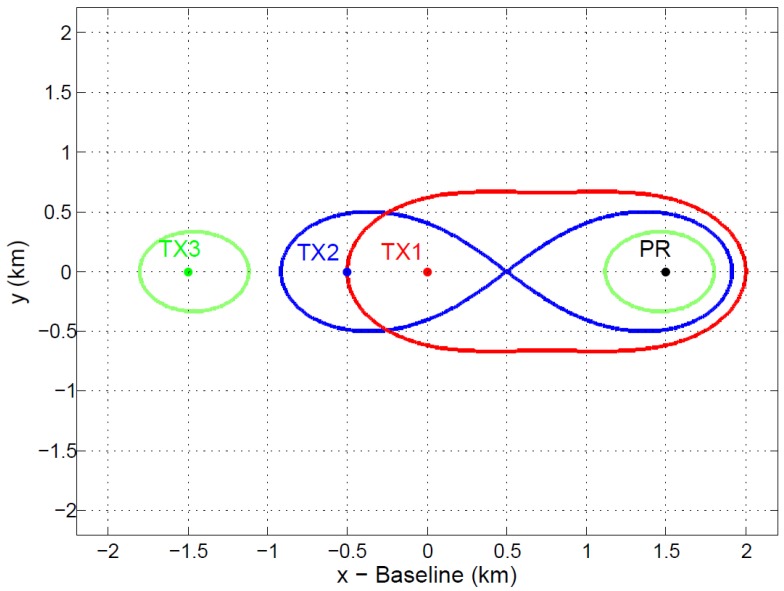
Study of different coverage areas for a fixed passive radar (PR) position as a function $L/(R_{T}R_{R})$ assuming $R_{T}\cdot R_{R}=1$ km${}^{2}$: $L/(R_{T}R_{R})=1.5$ in red, $L/(R_{T}R_{R})=2$ in blue and $L/(R_{T}R_{R})=3$ in green.

As an example of the impact of the $L/(R_{T}\cdot R_{R})$ relation on the coverage area geometry, a simple study is presented in Figure 7. Assuming a normalized value $R_{T}\cdot R_{R}=1$ km${}^{2}$, different coverage areas are represented for $L/\left(R_{T}\cdot R_{R}\right)\in \left\{1.5,2,3\right\}$.

### 2.5. Bistatic Radar Cross-Section

The bistatic RCS, $\sigma_{bis}$, is a function of the transmitted signal (frequency and polarization), the shape and materials of the target and the incidence and scattering directions. In the bistatic RCS region, characterized by bistatic angles higher than $5^{\circ }$ and lower than $180^{\circ }$, the bistatic RCS is usually lower than the monostatic RCS for complex targets. Exceptions include [27]:

1. Some target aspect angles that generate a low monostatic RCS and a high bistatic specular RCS at specific bistatic angles.
2. Targets that are designed for low monostatic RCS over a range of aspect angles.
3. Shadowing that sometimes occurs in a monostatic geometry and not in a bistatic one.

Taking into consideration the scenario geometry depicted in Figure 6 and the possible incidence and scattering directions, the monostatic and the bistatic RCSs of a car were estimated using the program POFACETS, developed at the Naval Postgraduate School [28]. Incidence and scattering directions are defined by pairs (θ,ϕ), where *θ* represents the elevation angle (measured with respect to the Earth’s surface perpendicularly) and *ϕ* the azimuth one (measured in the plane tangent to the Earth’s surface, with respect to the north). A basic car model made of perfect electrical conductor (PEC) with dimensions 6 m (L) × 2 m (W) × 1.6 m (H) was used, and the simulations were performed for f=9.65 GHz and vertical polarization. The RCS has dimensions of area, so an RCS value equal to $a(m^{2})$ corresponds to $A\left(dBsm\right)=10\cdot \log_{10}\left(a\right)$.

**Figure 8:**
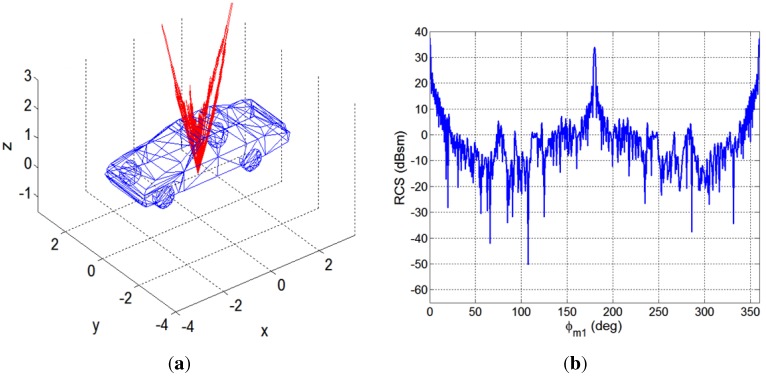
Results obtained for the Monostatic 1 case using POFACETS : (**a**) 3D model of the target and 3D monostatic radar cross-section (RCS); (**b**) Monostatic RCS in Cartesian coordinates.

**Figure 9:**
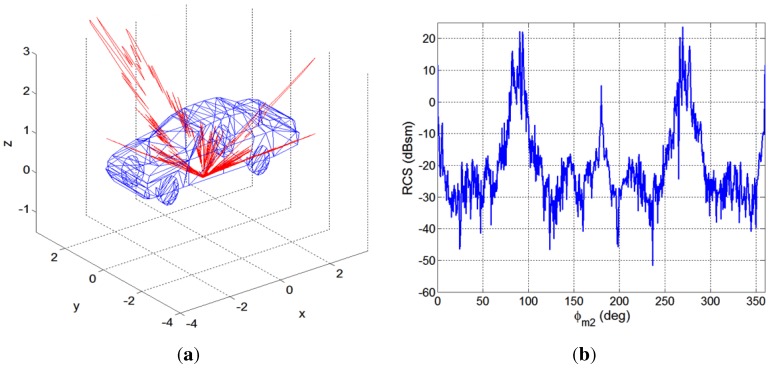
Results obtained for the Monostatic 2 case using POFACETS: (**a**) 3D model of the target and 3D monostatic RCS; (**b**) Monostatic RCS in Cartesian coordinates.

For the monostatic study, two cases were simulated: *Monostatic-1* ($\theta_{m1}=15^{\circ }$ and $\phi_{m1}\in \left[0,360^{\circ }\right]$), and *Monostatic-2* ($\theta_{m2}=65^{\circ }$ and $\phi_{m2}\in \left[0,360^{\circ }\right]$). In Figure 8 and Figure 9 the results obtained for the monostatic study cases, *Monostatic-1* and *Monostatic-2*, are depicted in 3D and in cartesian coordinates.

**Figure 10:**
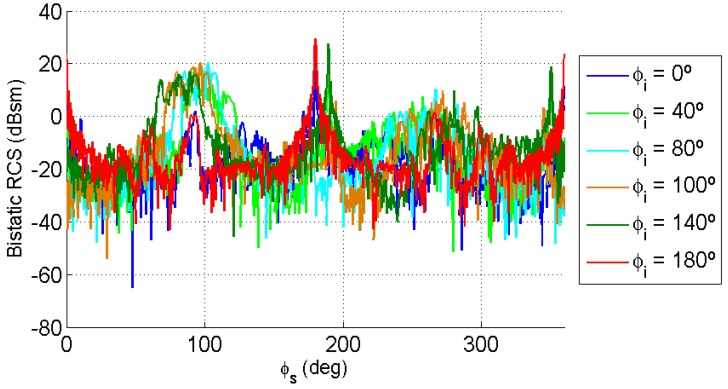
Bistatic RCS as a function of the scattering direction azimuth, $\phi_{s}$, for $\theta_{i}=15^{\circ }$, $\theta_{s}=90^{\circ }$ and $\phi_{i}\in \left\{0^{\circ },40^{\circ },80^{\circ },100^{\circ },140^{\circ },180^{\circ }\right\}$.

**Figure 11:**
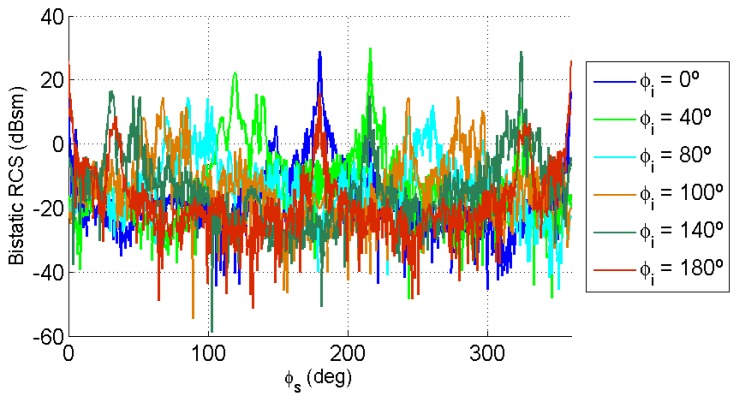
Bistatic RCS as a function of the scattering direction azimuth, $\phi_{s}$, for $\theta_{i}=65^{\circ }$, $\theta_{s}=90^{\circ }$ and $\phi_{i}\in \left\{0^{\circ },40^{\circ },80^{\circ },100^{\circ },140^{\circ },180^{\circ }\right\}$.

**Figure 12:**
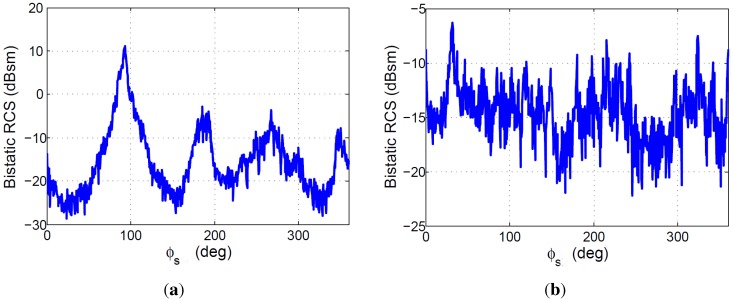
Bistatic RCS averaged along the incidence direction azimuth, $\phi_{i}$: (**a**) Bistatic 1 case study; (**b**) Bistatic 2 case study.

In the bistatic case, incidence and scattering directions must be considered. The estimation of an average bistatic RCS was performed using the following methodology:

- For each value of incidence elevation angle, $\theta_{i1}=15^{\circ }$ and $\theta_{i2}=65^{\circ }$, the incidence azimuth angle, $\phi_{i}$, was varied from $0^{\circ }$ to $180^{\circ }$, taking into consideration the symmetry of the car in the XY plane.
- For each incidence direction $\left(\theta_{i},\phi_{i}\right)$, the elevation angle of the scattered wave was fixed to $\theta_{s}=\;90^{\circ }$, and the azimuth scattering angle, $\phi_{s}$, was varied from $0^{\circ }$ to $360^{\circ }$. Figure 10 and Figure 11 show results obtained for $\theta_{i1}=15^{\circ }$ and $\theta_{i2}=65^{\circ }$, respectively.

- For each scattering direction, $\left(\theta_{s},\phi_{s}\right)$, and incidence elevation angle $\theta_{i}$, an average bistatic RCS was calculated as the mean value of the bistatic RCSs estimated for the set of $\phi_{i}$ values ranging from $0^{\circ }$ to $180^{\circ }$. The study carried out for $\theta_{i1}=15^{\circ }$ was denoted as *Bistatic-1* and the study for $\theta_{i2}=65^{\circ }$ was denoted as *Bistatic-2*. Results are presented in Figure 12.

Maximum, minimum and average values of the mean bistatic RCS along the $\phi_{s}$ dimension are summarized in Table 1. Results show that for the lower incidence angle (close to normal incidence), the monostatic RCS is higher than the bistatic one, but for higher values of the incidence angle, the bistatic RCS can be higher than the monostatic one.

**Table 1:** Bistatic RCS simulations results.

RCS	Monostatic 1	Bistatic 1	Monostatic 2	Bistatic 2
Maximum	37.385 dBsm	11.074 dBsm	23.611 dBsm	−6.293 dBsm
Minimum	−50.229 dBsm	−28.678 dBsm	−51.619 dBsm	−22.193 dBsm
Average	−3.331 dBsm	−15.522 dBsm	−20.532 dBsm	−14.658 dBsm

## 3. PAZ Description

The PAZ satellite platform is similar to the TerraSAR-X one and has been developed by Airbus DS. The instrument front-end has been designed by Airbus DS Spain/CASAEspacio ( Construcciones Aerospaciales S.A.). The Spanish National Institute for Aerospace Technology (INTA, Instituto Nacional de Tecnología Aerospacial) has been responsible for the development of the Ground Segment of the PAZ Mission, and it is responsible for PAZ scientific exploitation.

Scientific exploitation conveys all knowledge areas related to SAR technology: radio-frequency, digital signal processing, image processing, remote sensing, *etc*. The main objectives are the following:

- To identify areas of interest considering the Spanish environment and compromises.
- To provide data to the scientific community for educational, scientific and technological purposes.
- To research SAR systems’ characterization and calibration, operating modes’ definition, multi-static configurations and multi-sensor developments.
- To collaborate with high level SAR institutions.
- To develop a set of research-based demonstrators to prove SAR technology capabilities in different applications.

Searchers or research groups can communicate their interest to the e-mail address *PAZ_science@inta.es*.

For the proposed study, the antenna gain and the imaging modes are of great importance. They are analyzed in the following subsections.

### 3.1. PAZ Antenna

The PAZ antenna is an X-band active-phase planar array implemented in printed-radiator technology. Together with a low profile, low-mass, flexibility and easiness of manufacturing, this technology allows the development of flexible beamforming networks (BFN).

**Figure 13:**
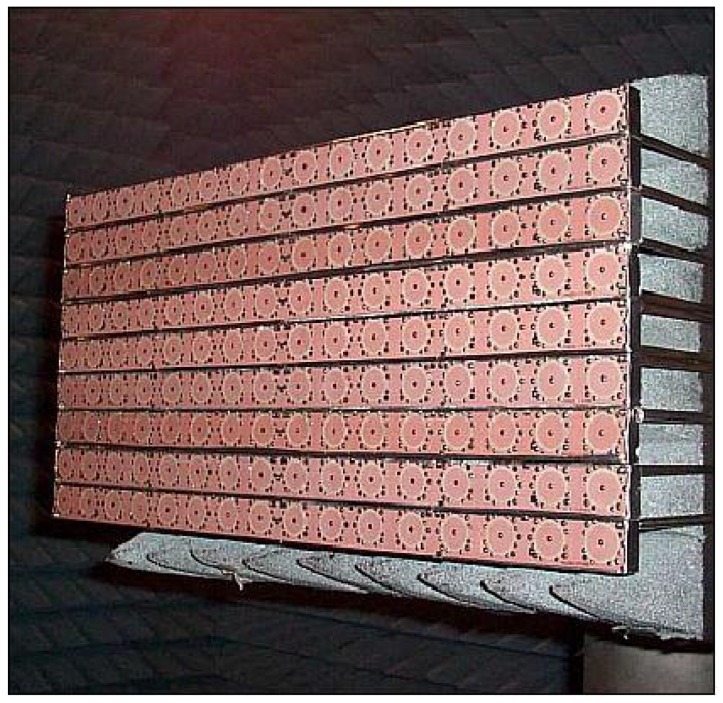
PAZ panel subarrays [8].

The PAZ planar array is composed of 12 panels (4.8 m × 0.7 m). Each panel is composed of 32 subarrays, and each subarray is composed of 16 microstrip patches. Each subarray is a single radiating element, which includes a dedicated transmit-receive module (TRM) adjustable in amplitude and phase by applying complex excitation coefficients. This enables beam steering, adaptive beamforming in the azimuth and elevation and the generation of more than ten thousand beams. An accurate antenna model (AMOR, Antenna MOdelleR ) was developed for generating the antenna patterns for improving radar image quality and calculating TRM settings to ensure the performance further on, even in the case of drifting and/or failed modules during the spacecraft lifetime, among other objectives [29]. Figure 13 shows a qualification model composed of nine subarrays [8].

The array gain is a key element in the feasibility study, so an estimated value was calculated using the available information [30]:

- Subarray directivity higher than 20.1 dB for vertical and horizontal polarizations.
- Losses lower than 1 dB.
- Peak power equal to 2.26 kW.

Assuming that all of the elements of the array are equal and unaffected by the position in the array, thus ignoring some possible edge effects, the array gain can be estimated as follows [31]:

(7) $$G_{array}\left(\theta ,\phi \right)=N\cdot D_{element}\left(\theta ,\phi \right)\cdot \epsilon_{element}\cdot L_{scan}$$

where:

- $\epsilon_{element}$ is the element efficiency term due to possible mismatching effects.
- $L_{scan}$ represents the scan loss effect, which is typically modeled as a function of $cos(\theta_{0})$, being $\theta_{0}$ the main beam pointing direction with respect to the array broadside direction.
- $D_{element}\left(\theta ,\phi \right)$ represents the directivity of each single radiating element.
- *N* is the number of single radiating elements in the array.

Given the similarities between PAZ and TerraSAR-X, the angle between the array panel inclination and nadir direction of TerraSAR-X was considered for the study, this value being equal to $33.8^{\circ }$.

Finally, the Equivalent Isotropic Radiated Power (EIRP) was estimated for *α* ranging from $15^{\circ }$ to $65^{\circ }$. For $\alpha =15^{\circ }$ and $\alpha =65^{\circ }$. EIRPs of 65.4 dBm and 63.4 dBm, were obtained, respectively.

### 3.2. Transmitted Signal

The PAZ signal is a periodic train of linear frequency-modulated pulses. The typical bandwidth will be 150 MHz, but it will be able to be increased to 300 MHz for special imaging modes. Table 2 summarizes the main signal parameters [32,33].

**Table 2:** PAZ signal parameters.

**Central frequency**	9.65 GHz
**Maximum bandwidth**	300 MHz
**Peak power**	1.9 kW
**Pulse length**	15 µs–67 µs
**Service factor**	18%–20%
**Pulse repetition frequency**	2 kH–6.5 kH

### 3.3. Orbit Parameters

PAZ will fly in a polar dawn-dusk Sun-synchronous orbit. This type of orbit allows power throughput maximization and simplifies the thermal design. TerraSAR-X and TanDEM-X satellites follow this same orbit, offering the capability for repeat-pass interferometry. PAZ will cover all of Earth with a mean revisit time of one day and a mean access delay slightly higher than 24 h (Table 3) [33].

**Table 3:** PAZ orbit and incidence angle values.

**Nominal height**	514 km
**Orbits per day**	15 + 2/11
**Incidence angle**	20${}^{\circ }$–45${}^{\circ }$ (full perf.) 15${}^{\circ }$–60${}^{\circ }$ (accessible)

### 3.4. PAZ Imaging Modes

**Figure 14:**
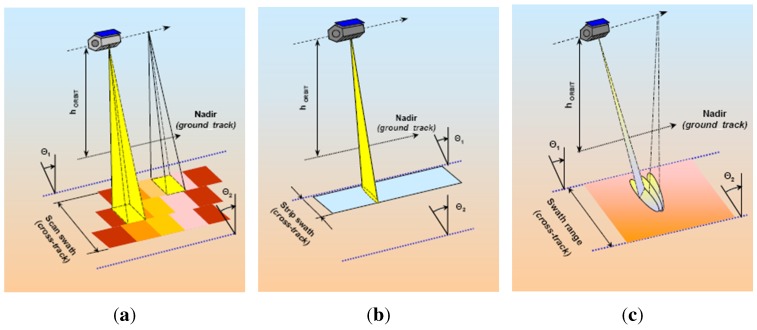
PAZ imaging models [8]: ScanSAR (**a**); stripmap (**b**); spotlight (**c**).

The PAZ satellite will operate in four nominal imaging modes (Figure 14):

- Stripmap: The antenna beam is pointed to a fixed angle in elevation and azimuth, resulting in a strip with constant quality in azimuth (azimuth resolution up to 3 m, scene size up to 30 km × 50 km). Single (HH, VV) and dual (HH/VV, HH/HV, VV/VH) polarization modes are possible.
- ScanSAR: The electronic antenna elevation steering is used to switch after bursts of pulses between swathes with different incidence angles. This mode has an azimuth resolution up to 18 m, with a scene size of 100 km × 150 km. It can only acquire images with single polarization (HH, VV).
- Spotlight: Azimuth phased array beam steering is used to increase the illumination time and the azimuth resolution, at the cost of azimuth scene size (azimuth resolution up to 2 m, scene size of 10 km × 10 km). Single (HH, VV) and dual polarization (HH/VV) operating modes are possible.
- High resolution spotlight: this mode has an azimuth resolution up to 1 m, with a scene size of 10 km × 5 km and is able to operate with single (HH, VV) and dual polarization (HH/VV).

New modes developed for TerraSAR-X, such as the staring spotlight and the wide ScanSAR, are foreseeable for PAZ.

## 4. Feasibility of PAZ as an Opportunity Illuminator in a Passive Bistatic Radar System

The feasibility study will be carried out taking into consideration several criteria: the signal waveform, the availability of the OI and the knowledge of its position, the signal power level and the instrumented coverage area.

### 4.1. OI Signal Waveform

Most of the detection capabilities of a passive radar system depend on the OI signal, in the same way that the dedicated transmitter determines the characteristics of active radars.

Bistatic range resolution depends on signal bandwidth and system geometry, as shown by expression Equation (3), so other criteria related to system geometry must be studied before facing the calculus of the bistatic range resolution.

For the waveform analysis, a synthetic signal was generated with the following parameters:

- Pulse repetition frequency, PRF = 3.03886 kHz
- Duty cycle, τ=19%.
- Pulse duration, T = 62.523 µs.
- Modulation factor, $k_{r}=2.0383\cdot 10^{12}$ Hz/s
- Bandwidth, ΔB=127.44 MHz
- Number of Linear Frequency Modulation (LFM) pulses, 50.

The ambiguity function is a powerful analysis tool for determining the detection capabilities of a waveform. It can be calculated using Equation (1), after substituting $s_{SURV}\left[n\right]$ by $s_{REF}\left[n\right]$.

**Figure 15:**
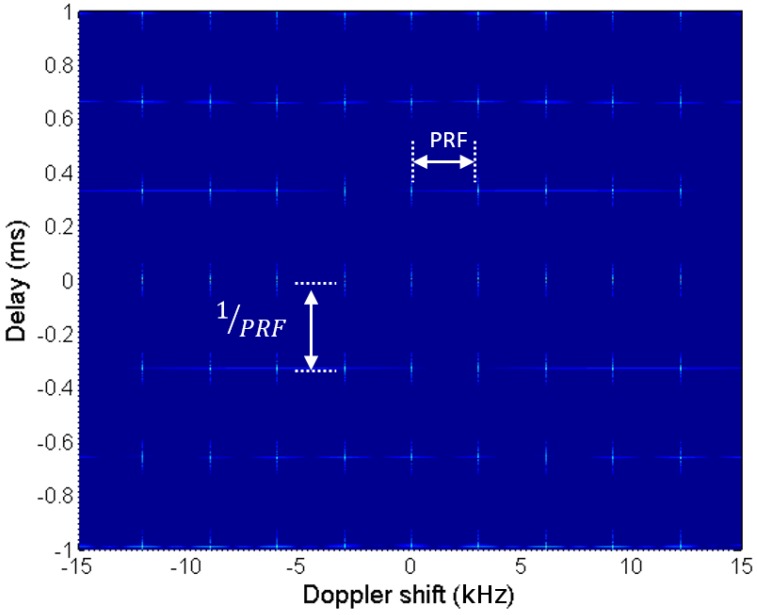
Ambiguity function (AF) estimation for a synthetic signal composed of a set of LFM pulses.

**Figure 16:**
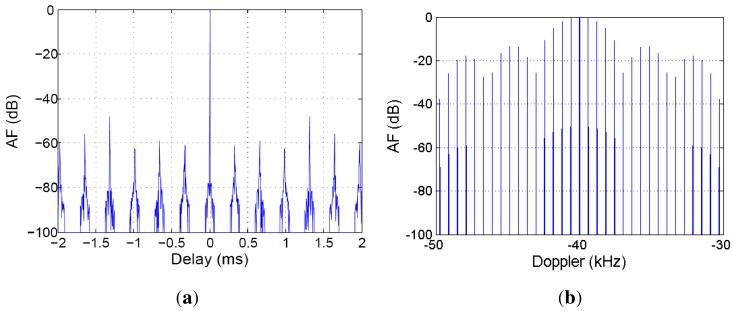
AF main cuts: (**a**) zero Doppler; (**b**) zero delay.

The AF of the synthetic signal is depicted in Figure 15 and Figure 16. The ambiguity peak pattern predicted in [34] can be clearly identified:

- Periodic ambiguity peaks appear along the time delay dimension, with a period equal to $\frac{1}{PRF}$.
- Periodic ambiguity peaks appear along the Doppler shift dimension, with a period equal to PRF.

These undesired peaks can mask desired targets or increase the probability of false alarms. To avoid these effects, radar coverage and desired target dynamics must be limited to guarantee the operation in the unambiguous region of the AF: time delay lower than $\tau =\frac{1}{PRF}=0.32908$ ms and Doppler shift lower than ν=PRF=3.03886 kHz.

### 4.2. OI Availability

The PR requires an OI that illuminates the area of interest. As discussed in Section 1, the availability constraints of an EO satellite are very tight, and are mainly related to its movement with respect to the Earth’s surface and its on-demand operation. Since no cooperation of the illuminator is required, other EO satellites could be used to increase the time an area is illuminated. As a matter of fact, both TerraSAR-X and TanDEM-X follow the same orbit of PAZ and have very similar characteristics. Furthermore, other SAR sensors, like the Cosmo SkyMed constellation, Sentinel-1 and the future Radarsat constellation, will also help to increase this time. A study of the potential bistatic geometry has to be carried out in order to determine parameters, such as:

- The time availability: determined by the sensor orbit parameters and the scheduled data acquisition processes. This study is directly related to the knowledge of the OI position that is studied in Section 4.3.
- The instrumented spatial coverage: determined by the EO satellite footprint, which depends on the sensor movement and acquisition mode. It is studied in Section 4.4.

### 4.3. Knowledge of the OI Position

**Figure 17:**
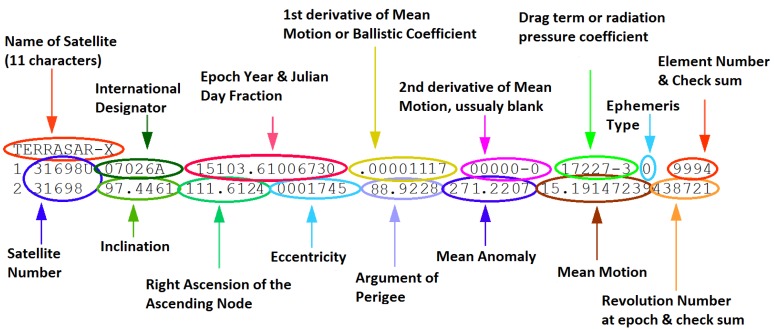
Two-line element (TLE) parameters for the TerraSAR-X orbit (13 April 2015) [36].

The position of the OI is a key element for the definition of the bistatic geometry. In Figure 6, $R_{T}+R_{R}$ can be estimated from the delay between the transmitted wave and the target echo, $\tau_{bistatic}$, and the baseline length, *L*, Equation (8). The instantaneous position of a satellite can be determined using the simplified general perturbation model (SGP4), which estimates the instantaneous two-line element (TLE) parameters of the orbit from an initial state [35]. The standard TLEs are routinely available from CelesTrak [36]. As an example, the TLE parameters of TerraSAR-X on 13 April 2015, obtained from that source, are presented in Figure 17.

(8) $$R_{T}+R_{R}=c\cdot \tau_{bistatic}+L$$

Given the instantaneous position of the OI, the location of the PR receiver and the radiation properties of the OI and the PR surveillance channel, the main geometry parameters ($R_{T}$, $R_{R}$ and *β*) can be estimated for each detected target as functions of time. A case study example is defined and analyzed in Section 5.

### 4.4. Instrumented Coverage Area

Due to the high distance between the OI and the PR receiver, $L>2(R_{T}R_{R})$, the coverage area is split into two ovals centered on the OI and the PR receiver, respectively. The one centered on the receiver is the coverage area to be exploited. The system area of interest must be located inside this oval.

The instrumented coverage area is determined by the OI antenna footprint. It depends on the selected imaging mode: stripmap, ScanSAR or spotlight. Due to the satellite and the SAR sensor antenna beam movement, the following issues must be considered:

- The reference channel must include tracking techniques to guarantee the reference signal reception.
- The surveillance channel must reject the OI direct signal (direct path interference (DPI)) as the OI beam moves. The PR receiver and the area of interest are in the same coverage oval, so both are illuminated by the OI. Targets to be sought are on the surface or at low altitudes compared to the OI position, making the rejection of the direct OI signal feasible (incidence angles in $\left[15^{\circ },65^{\circ }\right]$). The DPI rejection requires a surveillance antenna system capable of generating notches toward the satellite elevation and a receiver with properly-designed filtering techniques.
- If the OI antenna beam cannot be considered stationary or quasi-stationary during the acquisition time (stripmap or ScanSAR imaging modes), the coverage area will vary with the footprint movement.

**Figure 18:**
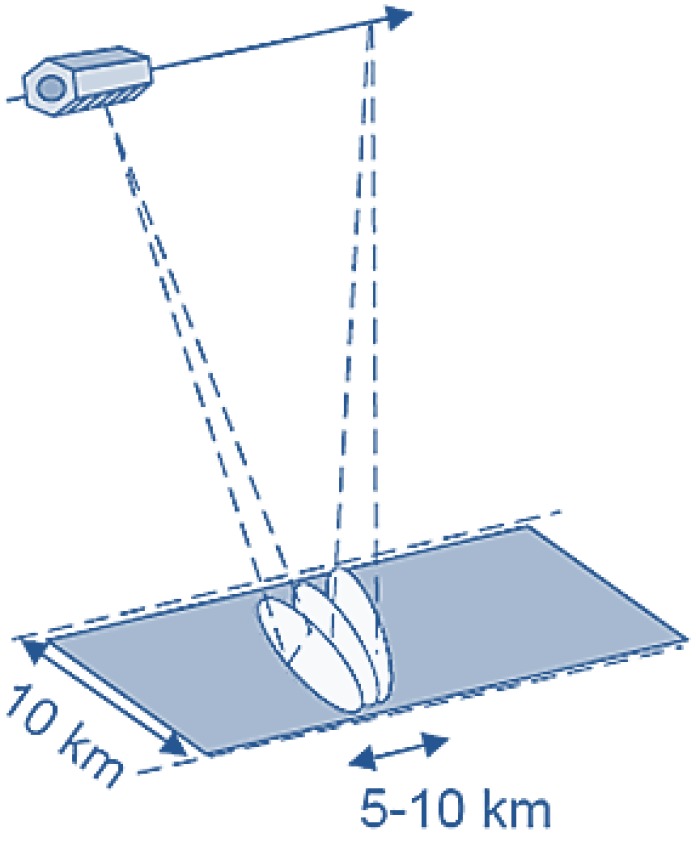
Footprint of the opportunity illuminator (OI) during the acquisition of a spotlight image.

In Section 5, a study of the system geometry is presented assuming a spotlight acquisition mode (Figure 18).

### 4.5. Incident Power Density and Required Sensitivity

For the study of the power density reaching the desired targets, the following considerations have been taken:

1. The incidence angle of the PAZ antenna beam, *α*, can vary in $\left[15^{\circ },65^{\circ }\right]$ (Figure 6). This parameter determines the baseline, the OI-target distances and the associated propagation losses.
2. The estimated values of Equivalent Isotropic Radiated Power (EIRP) calculated in Section 3.1 range from 65.4 dBm for $\alpha =15^{\circ }$, to 63.4 dBm for $\alpha =65^{\circ }$.
3. A receiver gain ($G_{R}$) of 23 dB was considered. Average bistatic RCS values were calculated for each incidence angle following the methodology explained in Section 2.5. Estimated values for the car model used in Section 2.5 are presented in Table 4.
4. The PR receiver was located at $40^{\circ }30^{\prime }48.44^{\prime \prime }$ N and $3^{\circ }20^{\prime }55.30^{\prime \prime }$ W.

**Table 4:** Simulated bistatic RCS for the car model used for calculating the received power in Figure 19.

RCS	$\alpha =15^{\circ }$	$\alpha =25^{\circ }$	$\alpha =35^{\circ }$	$\alpha =45^{\circ }$	$\alpha =55^{\circ }$	$\alpha =65^{\circ }$
Maximum	11.074 dBsm	−0.046 dBsm	−5.539 dBsm	−7.631 dBsm	−8.385 dBsm	−6.293 dBsm
Minimum	−28.678 dBsm	−29.317 dBsm	−28.164 dBsm	−26.995 dBsm	−26.685 dBsm	−22.193 dBsm
Average	−15.522 dBsm	−15.912 dBsm	−15.897 dBsm	−16.729 dBsm	−16.132 dBsm	−14.658 dBsm

**Figure 19:**
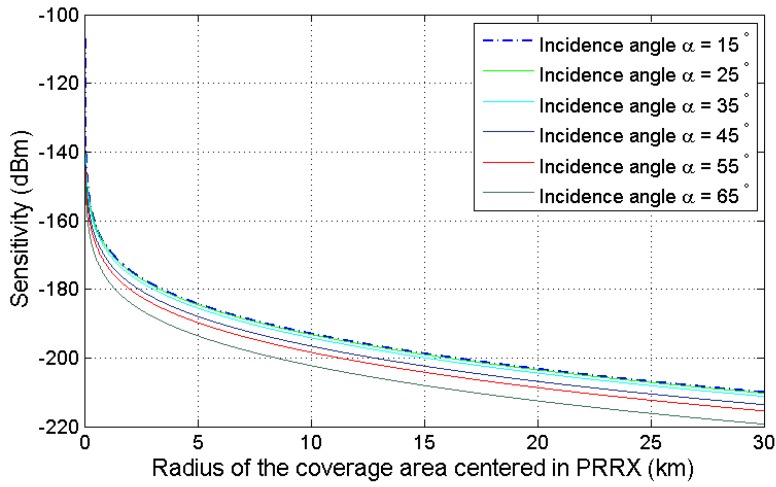
Coverage area of PAZ as the OI depending on the sensitivity of the system and the incidence angle of the satellite’s illumination beam.

The available power at the PR antenna was calculated as a function of the target-to-PR distance, and it is presented in Figure 19 as a function of the OI incidence angle. For a coverage of 15 km, affordable power levels are obtained, proving the feasibility of PAZ as the OI from the point of view of this parameter.

## 5. Case Study Example

The analysis of the bistatic geometry of a PR based on TerraSAR-X was carried out, because of the similar characteristics of PAZ and TerraSAR-X. The central point of the area of interest was located at the University of Alcalá campus, on the roof of the Polytechnic School, with the following coordinates $40^{\circ }30^{\prime }48.44^{\prime \prime }$ N, $3^{\circ }20^{\prime }55.30^{\prime \prime }$ W and an altitude of 587 m.

An acquisition in spotlight mode was assumed. This mode simplifies the geometry of the system, because it allows the definition of a constant coverage area during SAR sensor operation. It has been selected in order to focus on the description of the parameters to be considered in the analysis. Once the methodology has been explained, it can be straightforwardly applied to ScanSAR and stripmap modes, taking into consideration the movement of the footprint and the increase in the achievable coverage area.

**Figure 20:**
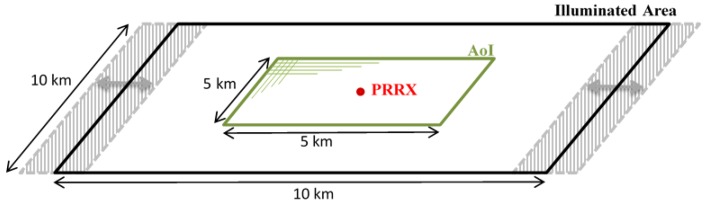
Case study geometry analysis scheme.

A spotlight image acquisition covering an area of 10×10 km${}^{2}$, with a bandwidth of 300 MHz, an incidence angle of $24.75^{\circ }$, and an acquisition time of 1.5 s was assumed (Figure 18). Due to the slight movement of the beam along the cross-range direction, an effective inner illuminated area of 5×5 km${}^{2}$ was considered (Figure 20).

The PR receiver was located at the center of the area of interest. This solution minimized the required sensitivity, taking into consideration that, as has been already mentioned, the coverage was composed of two independent ovals centered on the OI and the PR receiver, respectively. The considered area laid completely in the unambiguous detection region defined by the LFM pulse train CAF, which is around 480 km long for the monostatic case.

The date and time parameters of the case of study were: 13 April 2015, 17:58:50. From the TLE parameters provided by the SGP4 model, during the acquisition time of 1.5 s, the main features of the satellite movement where estimated:

- The satellite moved from $40^{\circ }10^{\prime }20.00^{\prime \prime }$ N, $5^{\circ }51^{\prime }56.00^{\prime \prime }$ W to $40^{\circ }15^{\prime }57.00^{\prime \prime }$ N, $5^{\circ }53^{\prime }24.00^{\prime \prime }$ W.
- Satellite altitude remained approximately constant and equal to 513.4 km.
- The acquisition started at the time when the elevation angle of the satellite with respect to the PR receiver location was maximum. The elevation variations were of some hundredths of degree around $65.25^{\circ }$.
- The azimuth of the satellite with respect to the North varied $3^{\circ }$, from $260^{\circ }$ to $263^{\circ }$. This variation was higher due to the polar orbit of the sensor.

Knowing the position of the OI during the acquisition time, a bistatic system geometry analysis was carried out throughout the coverage area (Figure 20) with the following considerations:

- The area of interest was divided into 5×5 m${}^{2}$ cells.
- The system geometry was calculated assuming that a non-moving ground target was located at the center of each cell.
- The system geometry was recalculated each 0.01 s (150 time slots were analyzed) in order to take into account the satellite dynamics.

**Figure 21:**
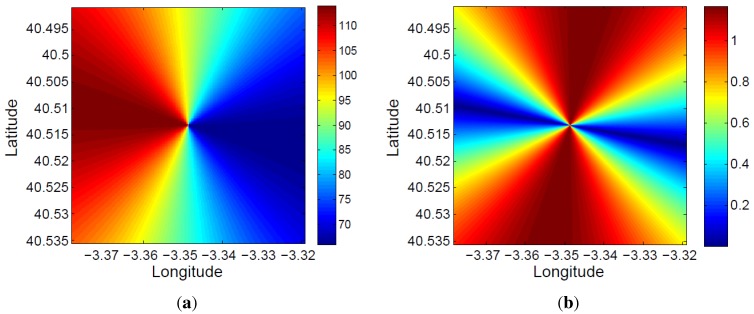
Study of the spatial and time variation of the bistatic angle: (**a**) initial value at each 5×5 m${}^{2}$ cell of the coverage area; (**b**) total variation during the acquisition time.

The bistatic angle at the acquisition starting time for each 5×5 m${}^{2}$ cell of the defined coverage area is presented in Figure 21a. The total variation of the bistatic angle during the whole acquisition time is represented in Figure 21b. The maximum bistatic angle variations is equal to $1.17^{\circ }$.

**Figure 22:**
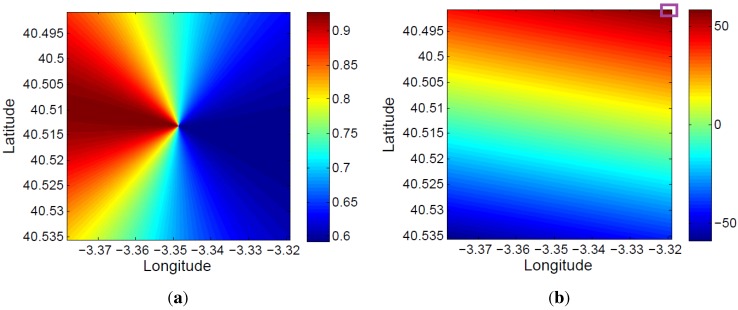
Bistatic range resolution and bistatic range: (**a**) initial bistatic range resolution at each 5×5 m${}^{2}$ cell of the coverage area; (**b**) total variation of the bistatic range during the acquisition time.

The bistatic range resolution, which is a function of the bistatic angle, was also analyzed. Using Equation (3), its value at the acquisition starting time for each 5×5 m${}^{2}$ cell of the defined coverage area was calculated (Figure 22a). Due to the small variation of the bistatic angle during the acquisition time, the variation of the bistatic range resolution in the 1.5 s has not been represented.

Because of the satellite movement, the bistatic time delay and the associated bistatic range are also a function of time ($R_{T}+R_{R}-L$, in Figure 6). This makes the estimated target location a time-dependent variable. In Figure 22b, the total variation of the bistatic range during the whole acquisition time is represented for each 5×5 m${}^{2}$ cell of the defined area of coverage. In this case, variations of 50 m are calculated.

**Figure 23:**
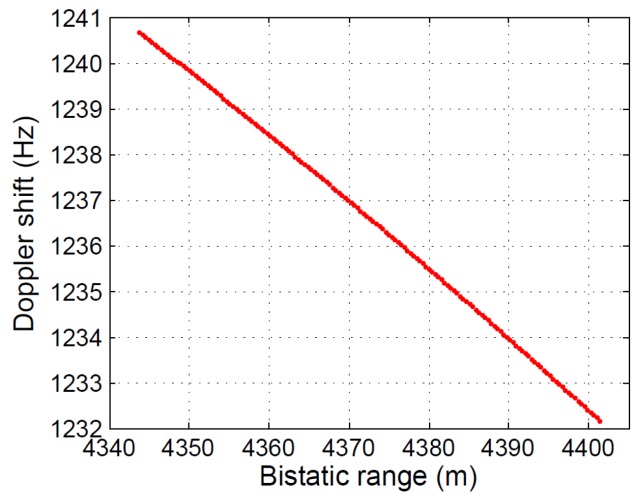
Virtual displacement for a fixed point inside the area of interest.

The variation of the bistatic range with time for a stationary target (*i.e.*, corner reflector) located at the center of a 5×5 m${}^{2}$ cell gives rise to the virtual movement of the stationary target. In Figure 23, the virtual trajectory of a stationary target located at the upper right corner cell of Figure 22b is depicted. During the acquisition time, an average speed of 38 m/s and a total variation of the bistatic range of around 55 m were estimated.

**Figure 24:**
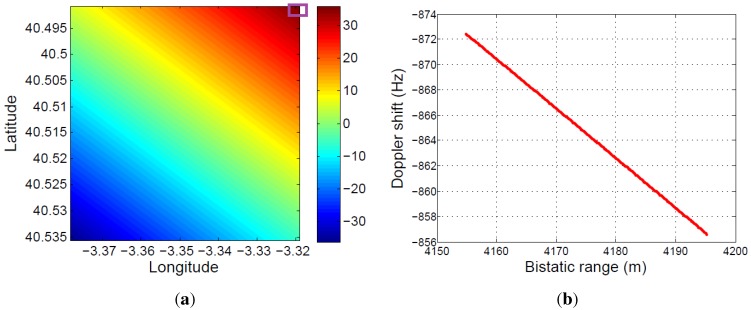
System geometry analysis assuming different TerraSAR-X orbital parameters (3 June 2015, 17:34:15): (**a**) total variation of the bistatic range during the acquisition time; (**b**) virtual displacement associated with satellite movement.

**Figure 25:**
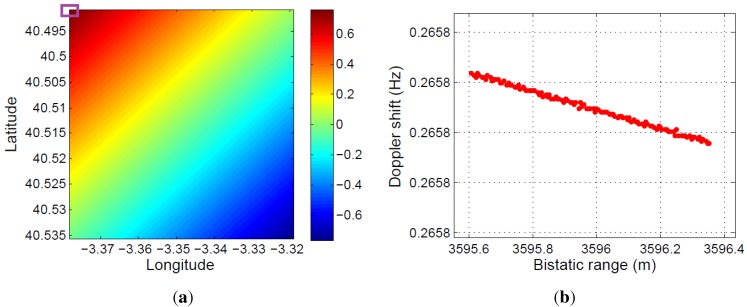
System geometry analysis for Navstart 59 (USA 192), on 25 May 2015, at 12:20:00: (**a**) total variation of the bistatic range during the acquisition time; (**b**) virtual displacement associated with the satellite orbit.

This virtual movement is completely defined by the system geometry, so it can be predicted and, therefore, compensated. To complete the study, two examples of different system geometries are presented:

- Virtual movement analysis for a bistatic radar scenario using the same satellite, but assuming a different acquisition date: The new date and time parameters are: 3 June 2015, 17:34:15. Figure 24 shows the total variation of the bistatic range and the virtual movement of the same stationary target as in the previous study, during the same acquisition time. In this case, the maximum magnitude of the bistatic range variation is approximately 35 m, lower than the approximated 55 m obtained in the previous study.

- Virtual movement analysis for a bistatic radar scenario using a GPS satellite: A new study case is considered exploiting the signal transmitted by Navstart 59 (USA 192). This is a medium-orbit satellite. The date and time parameters are: 25 May 2015, 12:20:00. The characteristics of the orbit and the transmitted signal parameters used in the study are summarized in Table 5 and Table 6, respectively. Results are presented in Figure 25. In this case, the stationary target is assumed to be located on the upper left corner of Figure 25a, in order to perform the analysis for a case characterized by a big variation of the considered parameters. For the same acquisition time used in previous examples, the obtained virtual displacement is significantly lower, less than 1 m.

These results validate the proposal of a PAZ-based PR for PAZ calibration and maintenance purposes and for surveillance of critical areas where the increase of monitoring time and/or target speed estimation could be important added values.

**Table 5:** GPS signal parameters used in the third geometry study.

**Central frequency**	1557 MHz
**Bandwidth**	2 MHz
**Power**	27 W
**Modulation**	Spread spectrum
**Transmission rate**	50 bps

**Table 6:** GPS orbit parameters.

**Nominal height**	20,200 km
**Orbits per day**	2

## 6. Conclusions

PAZ, the satellite EO SAR sensor of the Spanish National Earth Observation Program, is a powerful observation tool that will be used in the different applications defined in Copernicus, the European Earth observation program: land monitoring, marine monitoring, emergency management and security.

Taking into consideration the increasing interest in passive radars and the potential good features of a system such as PAZ, a complete study about PAZ’s feasibility as an opportunity illuminator in passive radar applications was carried out. Bistatic SAR systems have been the object of study, and some works dealing with the generation of passive radar images have been published. In this paper, the feasibility of PAZ as an opportunity illuminator in a passive bistatic radar system in surveillance applications was considered. Although a major drawback of that system is its intermittency in acquisition, constrained by the orbit parameters and its on-demand operating principle, PAZ is a powerful detection tool, with a finite lifetime, factors that justify the search for alternative applications.

A detailed study was carried out to analyze the influence of waveform, platform movement, variable bistatic geometry, transmitted power and on-demand operation mode. The main conclusions are the following:

- The transmitted signal is characterized by periodic ambiguity peaks that limit the coverage area and the target dynamics. After a study of instrumented coverage and system sensitivity, the limits imposed by the signal AF are beyond the limits imposed by these other factors, so the ambiguity peaks have no practical effect on system performance.
- OI availability: A study of potential bistatic geometries was carried out in order to determine the time availability and the instrumented spatial coverage. The orbital parameters, the acquisition modes and the operation schedule impose critical limitations on the OI availability. Taking into consideration these limitations, the following potential applications are proposed:
  − A PR system exploiting the signal emitted by PAZ can be used as a low-cost, easily deployable and configurable calibration tool in the commissioning phase of the SAR sensor or in posterior maintenance processes. The cost associated with the PR receiver is expected to be reduced due to the availability of commercial systems for direct receiving of sensor X-band data.
  − PR based on PAZ can also provide additional information, especially that related to the speed and trajectory of moving targets, to improve the information extraction from the acquired SAR images in the operational phase. This application can be really important in areas of special interest for monitoring specific phenomena.
  − As a PR can exploit the signal transmitted by other SAR sensors, GNSS and communication satellites, BSAR images can be generated to fill time gaps during which PAZ is not available.
- Instrumented coverage area: This is determined by the OI antenna footprint. The spotlight acquisition mode simplifies the geometry of the system, because it allows the definition of a constant coverage area during SAR sensor operation. For stripmap or ScanSAR operation, the knowledge of the sensor position makes it possible to study the movement of the coverage area, providing an increase of the achievable coverage.
  Different study cases were defined to analyze the impact of OI orbit: two study cases based on TerraSAR-X with different orbit parameters and a study case based on a GPS satellite.
- Incident power density and required sensitivity: The available power at the PR antenna was estimated as a function of the target-to-PR distance, proving the feasibility of PAZ as an IO from this point of view, allowing coverages of 15 km with affordable system sensitivities.

## References

[1] Willis N.J. (2005). Bistatic Radar.

[2] Cristallini D., Caruso M., Falcone P., Langellotti D., Bongioanni C., Colone F., Scafe S., Lombardo P. Space-Based Passive Radar Enabled by the New Generation of Geostationary Broadcast Satellites. Proceedings of the IEEE Aerospace Conference.

[3] Glennon E., Dempster A., Rizo C. (2006). Feasibility of Air Target Detection Using GPS as a Bistatic Radar. J. Glob. Position. Syst..

[4] Barcena-Humanes J.L., del Rey-Maestre N., Jarabo-Amores M., Mata-Moya D., Gomez-del Hoyo P. Passive radar imaging capabilities using space-borne commercial illuminators in surveillance applications. Proceedings of the Signal Processing Symposium (SPSympo).

[5] Pierdicca N., De-Titta L., Pulvirenti L., Della-Pietra G. (2009). Bistatic Radar Configuration for Soil Moisture Retrieval: Analysis of the Spatial Coverage. Sensors.

[6] Copernicus European Earth Observation Program. http://www.copernicus.eu/.

[7] Maillard P., Alencar-Silva T., Clausi D. (2008). An Evaluation of Radarsat-1 and ASTER Data for Mapping Veredas (Palm Swamps). Sensors.

[8] Earth-Observation-Portal. eo Sharing Earth Observation Resources “PAZ SAR Satellite Mission of Spain”. https://directory.eoportal.org/web/eoportal/satellite-missions/p/paz.

[9] Breit H., Fritz T., Balss U., Lachaise M., Niedermeier A., Vonavka M. (2010). TerraSAR-X SAR Processing and Products. IEEE Trans. Geosci. Remote Sens..

[10] Rodriguez-Cassola M., Prats P., Schulze D., Tous-Ramon N., Steinbrecher U., Marotti L., Nannini M., Younis M., Lopez-Dekker P., Zink M. (2012). First bistatic spaceborne SAR experiments with TanDEM-X. IEEE Geosci. Remote Sens. Lett..

[11] Dubois-Fernandez P., Cantalloube H., Vaizan B., Krieger G., Horn R., Wendler M., Giroux V. (2006). ONERA-DLR bistatic SAR campaign: Planning, data acquisition, and first analysis of bistatic scattering behavior of natural and urban targets. IEE Proc. Radar Sonar Navig..

[12] Antoniou M., Cherniakov M. (2013). GNSS-based bistatic SAR: A signal processing view. EURASIP J. Adv. Signal Process..

[13] Chai S., Chen W., Chen C. (2014). Sparse Fusion Imaging for a Moving Target in T/R-R Configuration. Sensors.

[14] Walterscheid I., Espeter T., Klare J., Brenner A. Bistatic Spaceborne-Airborne Forward-looking SAR. Proceedings of the 2010 8th European Conference on Synthetic Aperture Radar (EUSAR).

[15] Rodriguez-Cassola M., Baumgartner S.V., Krieger G., Moreira A. (2010). Bistatic TerraSAR-X/F-SAR spaceborne-airborne SAR experiment: Description, data processing, and results. IEEE Trans. Geosci. Remote Sens..

[16] Sanz-Marcos J., Lopez-Dekker P., Mallorqui J.J., Aguasca A., Prats P. (2007). SABRINA: A SAR bistatic receiver for interferometric applications. IEEE Geosci. Remote Sens. Lett..

[17] Lopez-Dekker P., Mallorqui J.J., Serra-Morales P., Sanz-Marcos J. (2008). Phase synchronization and Doppler centroid estimation in fixed receiver bistatic SAR systems. IEEE Trans. Geosci. Remote Sens..

[18] Lombardo P., Sedehi M., Colone F. (2007). Multi-Channel SAR Experiments from the Space and from Ground: Potential Evolution of Present Generation Spaceborne SAR.

[19] Yarman C., Yazici B. (2008). Synthetic Aperture Hitchhiker Imaging. IEEE Trans. Image Process..

[20] Wang L., Yarman C., Yazici B. (2011). Doppler-Hitchhiker A Novel Passive Synthetic Aperture Radar Using Ultranarrowband Sources of Opportunity. IEEE Trans. Geosci. Remote Sens..

[21] Maslikowski L., Samczynski P., Baczyk M., Krysik P., Kulpa K. (2014). Passive bistatic SAR imaging-Challenges and limitations. IEEE Aerosp. Electron. Syst. Mag..

[22] Maslikowski L., Samczynski P., Baczyk M. X-band receiver for passive imaging based on TerraSAR-X illuminator. Proceedings of the Signal Processing Symposium (SPS).

[23] Krysik P., Maslikowski L., Samczynski P., Kurowska A. Bistatic ground-based passive SAR imaging using TerraSAR-X as an illuminator of opportunity. Proceedings of the 2013 International Conference on Radar (Radar).

[24] Behner F., Reuter S. HITCHHIKER—Hybrid Bistatic High Resolution SAR Experiment using a Stationary Receiver and TerraSAR-X Transmitter. Proceedings of the 2010 8th European Conference on Synthetic Aperture Radar (EUSAR).

[25] Behner F., Reuter S., Nies H., Loffeld O. Synchronization and Preprocessing of Hybrid Bistatic SAR Data in the HITCHHIKER Experiment. Proceedings of the EUSAR 2014 10th European Conference on Synthetic Aperture Radar.

[26] Reuter S., Behner F., Nies H., Loffeld O., Matthes D., Schiller J. Development and experiments of a passive SAR receiver system in a bistatic spaceborne-stationary configuration. Proceedings of the 2010 IEEE International Geoscience and Remote Sensing Symposium (IGARSS).

[27] Skolnik M. (2008). Radar Handbook.

[28] Jenn D.C. Radar Cross Section Calculations Using the Physical Optics Approximation, POFACETS. http://faculty.nps.edu/jenn/.

[29] Sanchez Palma J., Solana Gonzalez A., Martin Hervas I., Monjas Sanz F., Labriola M., Martinez Cengotitabengoa J., Garcia Molleda F.M., Moreno Aguado S., Saameno Perez P., Closa Soteras J. SAR Panel Design and Performance for the PAZ Mission. Proceedings of the 2010 8th European Conference on Synthetic Aperture Radar (EUSAR).

[30] Astium C.E.E.P.T. Paz Instrument Front-End. Description and performance. Proceedings of the INTA Conference on SAR Technologies and Applications.

[31] Mailloux R.J. (2005). Phased Array Antenna Handbook.

[32] Primo M.C. (2011). Programa PAZ. Solución Española Observación todo Tiempo PAZ program. Anytime Observation Spanish Solution. Technology and Applications of Synthetic Aperture Radar (SAR).

[33] Gómez B., Gonzalez M.J., Braeutigam B., Vega E., Garcia M., Casal N., del Castillo J., Cuerda J.M., Alfaro N., Alvarez V. PAZ Mission: CALVAL Centre Activities. Proceedings of the European Synthetic Aperture Radar Conference.

[34] Levanon E.M. (2004). Radar Signals.

[35] Vallado D.A., Crawford P. SGP4 orbit determination. Proceedings of the AIAA/AAS Astrodynamics Specialist Conference and Exhibit.

[36] Celestrak Center for Space Standards and Innovation. https://celestrak.com.

